# African multilingualism viewed from another angle: Challenging the Casamance exception

**DOI:** 10.1177/13670069211023146

**Published:** 2021-12-06

**Authors:** Serge Sagna, Abbie Hantgan

**Affiliations:** University of York, UK; LLACAN, CNRS, France

**Keywords:** African multilingualism, monolingual language use, child language acquisition

## Abstract

**Aims and Objectives/Purpose/Research Questions:**

The former region of southern Senegal, the Casamance, has been portrayed throughout the literature on African multilingualism in a singular light, for example, as an area where monolingualism does not exist. The purpose of this article is to stress the previously unacknowledged importance of monolingual settings and practices by discussing data that have yet to be presented in the literature.

**Design/Methodology/Approach:**

We investigate rural multilingualism and monolingualism across the Casamance by carrying out the following four studies: (a) we conduct a survey of 62 villages with a questionnaire and our newly created ‘blindfold test’, classifying them into two main types; (b) with 34 women we study the role of exogamy in multilingual language acquisition in one of the villages; (c) we analyse child language production data and child directed speech to examine the existence of monolingual language acquisition; (d) we examine the sociolinguistic profiles of 101 speakers of one language community to investigate intergenerational multilingualism.

**Data and Analysis:**

Data were analysed using descriptive statistics in the form of frequency counts. Additionally, we couch our results on multilingualism in the theory of canonical typology.

**Findings/Conclusions:**

We propose a distinction between multilingual settings, e.g. communities where speakers are most likely to accommodate, and who live among villages largely located on national roads and around cities, and monolingual settings, which constitute most of the villages of the Casamance and where language acquisition is monolingual and where migration, rather than exogamy, accounts for the development of individual multilingualism.

**Originality:**

This article contributes unprecedented research methodology for the study of complex multilingual situations such as those found in African multilingual contexts.

**Significance/Implications:**

Our study adds to the growing understanding of small-scale multilingualism and the emergence of multilingualism in monolingual contexts.

## Introduction

**Figure 1.**
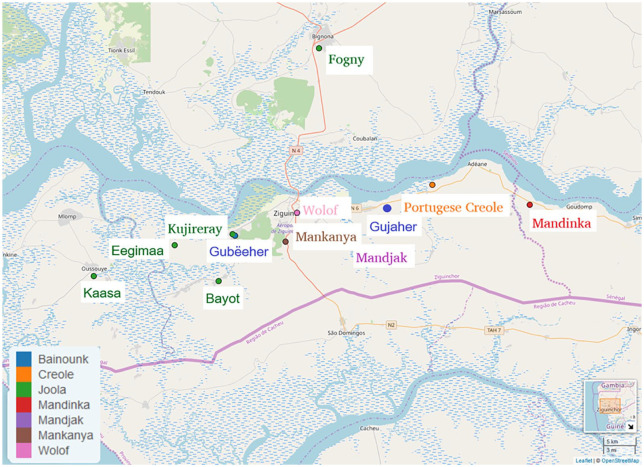
Casamance languages implicated in this study, colored by languages or language group. Map produced with Lingtypology package (Moroz, 2017) for R (R Core Team, 2017).

The Casamance is an area in southwestern Senegal that has a rich cultural heritage and has been of great interest to researchers from many different perspectives. Linguistically, the Casamance is home to a plethora of languages, 11 of which are illustrated by the map in Figure 1. Naturally, the map cannot accurately encompass the situation of language contact in the Casamance, and these points are merely an approximation of the areas in which they are spoken.

**Figure 2.**
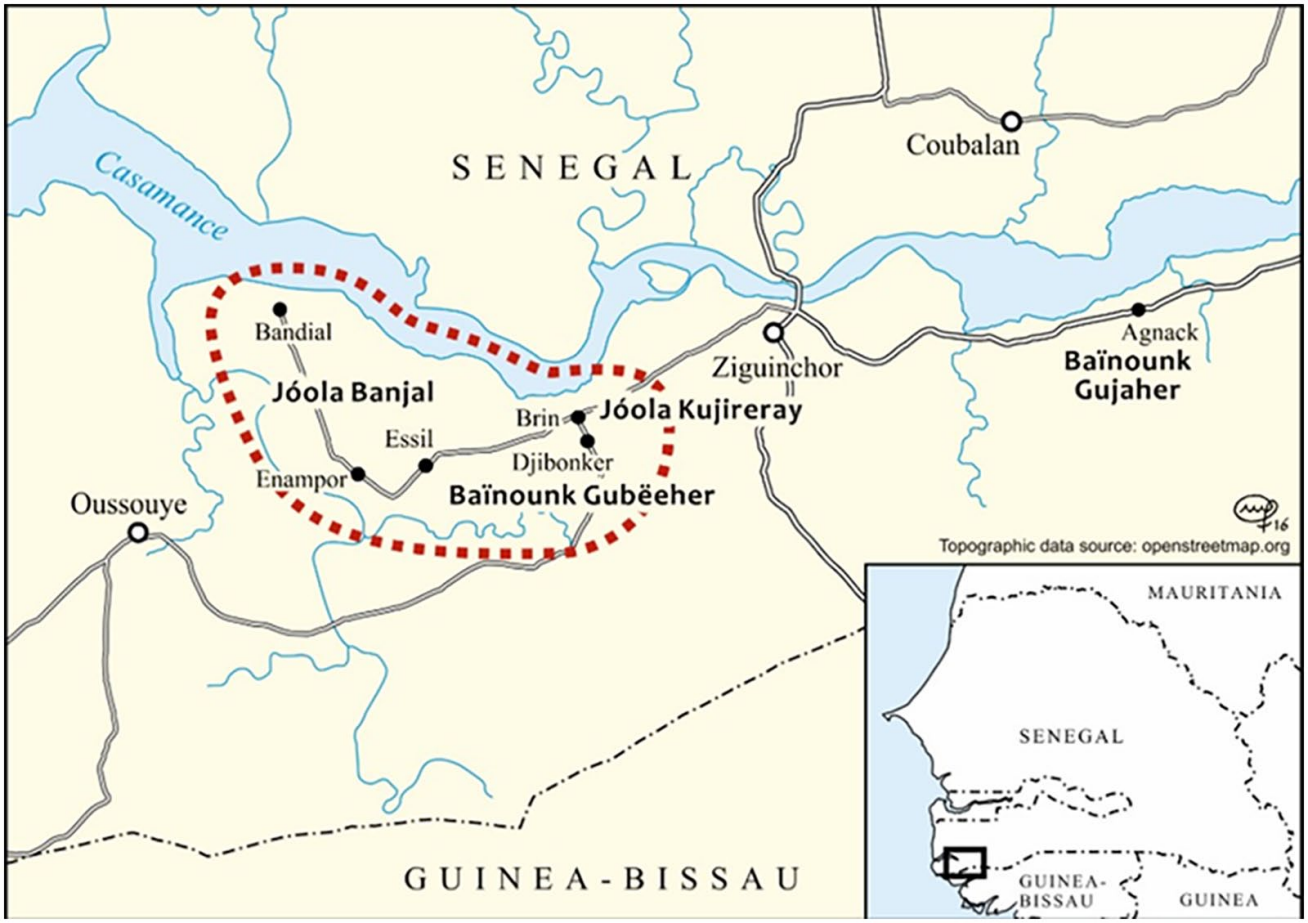
Crossroads area within dotted circle (source: www.soascrossroads.org).

There is a general agreement among researchers that the Casamance area has a large concentration of languages which can be estimated at around 20 or more, depending on how languages and dialects are distinguished. The prominent languages in our discussion include Eegimaa, also known as Jóola Banjal as in the map in Figure 2, which is a language of the Jóola cluster of the Atlantic branch, Jóola Foñi (spoken on the northern bank of Casamance River), the main Jóola lingua franca, Jóola Kaasa, the second main Jóola lingua franca, spoken along the southern bank of the Casamance River, and Kujireray, a Jóola language whose speakers are in close contact with those of the Baïnounk language, Gubëeher. Baïnounk languages are also classified as Atlantic languages, as is Wolof, which is the primary language of wider communication spoken by over 90% of the Senegalese population. Wolof is a prestigious language, especially among the younger speakers, and is associated with a fashionable way of life, as well as being the main language of dating among the youth. Other Atlantic languages relevant to our discussion include Jóola Bayot, Manjack, and Mankanya. Mandinka is from the Mande language family, which is remotely related to the Atlantic languages, but is found in some of the repertoires of the speakers we have interviewed. Finally, French, the official language of Senegal, is very present in speakers’ repertoires, especially those who have had formal education.

The most recent study focusing on languages of the Casamance is the Leverhulme Trust Research Leadership Award funded Crossroads Project,^1^ which took place from January 2014 to December 2018. The study focused on a point at which a group of three villages, each with nominally different languages met, and is hence titled The Crossroads. The project also encompassed a separate village, Agnack, which was said to serve as a control village. The map in Figure 2 shows the positions of the villages studied during the Crossroads Project.

However, there are conflicting views on how to understand and account for multilingualism in the Casamance, southern Senegal (Lüpke, 2016a; Sagna, 2016). In this article, we argue following Sagna & Bassène (2016) (see also Sagna, 2016) that to fully understand the current multilingual situation of the Casamance, a distinction must be made between multilingual versus monolingual settings, and individual multilingualism versus societal multilingualism. These concepts are discussed in Background, the next section, where we also provide a depiction of the existing literature and language situation in Casamance relevant to the study presented in this article. In the third section we present the materials and methods used in our research. We base our understanding of multilingualism in the Casamance on both our research and life experience. The results of our data collection are provided in the fourth section, while in the fifth we discuss our findings. The Conclusion, the sixth section highlights avenues for future research.

## Background

### Background of the relevant literature, villages and languages

The complex multilingual situation of the Casamance, the former administrative region of southern Senegal, has been presented in recent work as typologically exceptional, where individual and societal monolingualism are seen as fictional, if they exist at all. Specifically, it has been claimed that ‘there is no such thing as monolingual language acquisition in Senegal’ (Lüpke, 2016a, p. 10) and ‘every single household in Casamance being factually multilingual’ (Lüpke, 2016b, p. 50). Research that recognises monolingual settings are often described as being part of an ancestral code (Goodchild, 2018), or reflecting some form of essentialism or colonial language ideologies (Lüpke, 2016a).

An important argument we wish to make in this article is that the concentration of languages differs from one village (or set of villages) to another, and that this accounts for the intensity of language contact and the multilingual situation. Of the four villages included in the Crossroads Project (see map, Figure 2), two are Baïnounk villages (Djibonker and Agnack), one village, Brin, is contiguous to Djibonker, and is inhabited by Jóola people whose language, Kujireray (Watson, 2015) has a longstanding contact with Baïnounk Gubëeher from Djibonker. Baïnounk people are known to be more accommodating than other ethnic groups (Ducos, 1980), which, we argue, accounts for the higher rate of multilingualism in their villages and among individual speakers.

The studies where we conducted our research are concentrated in a former kingdom of 10 villages called Mof-Ávvi (the area where Jóola Banjal – also known as Jóola Eegimaa – is spoken as shown on the map in Figure 2), located to the west of Ziguinchor, the main city of the administrative region of Ziguinchor, in the Lower Casamance. One of these 10 villages, Essil, is the village of origin of the first author, who has been doing fieldwork there and in the other nine villages since 2003. Essil was also included in the Crossroads Project (Goodchild, 2018). Mof-Ávvi is included among our monolingual settings and its language of communication is Eegimaa. What is striking for our monolingual settings is that, presently, they include more multilingual than monolingual speakers. How it is possible to have multilingual speakers in what we refer to as monolingual areas will be clarified below.

### Multilingualism and monolingualism: A terminological background

In this article, we make a distinction between *societal multilingualism* and *individual multilingualism*. In the context of the Casamance, societal multilingualism refers to the language situation in a town, village or district, or a settlement where more than one language is not only required, but also used, in everyday communication. As detailed in the following subsection, ‘Multilingual settings’, this would be a place whose inhabitants need more than one language to successfully communicate with each other. The opposite, societal monolingualism, discussed further in the following subsection, ‘Monolingual settings’, refers to a settlement where only one default language is required and is used between residents to communicate. In our view, individual multilingualism refers to a person’s ability to communicate (beyond simple phrases, e.g. greetings and directions) in more than one language. Despite this, for the purposes of the current study, we include persons with a passive knowledge of another language in our individual multilingual category. Given the difficulty of determining the exact level of speakers’ multilingualism, we do this to avoid biasing our results towards monolingualism.

As discussed below, we use the term individual monolingualism to describe a person whose communicative abilities are restricted to one language. Having a simple repertoire of, for example, only greetings in another language, is not enough, in the view taken here, to define an individual as multilingual. We detail these arguments further with ideas from canonical typology in the subsection titled ‘Assessing monolingualism from a canonical viewpoint’. These concepts, which are briefly introduced here and discussed further in the next subsections, are important in understanding the contrast we make between multilingual and monolingual settings in the Casamance. They are also crucial to understanding at what level, or in what context it has been argued that, ‘[in] Africa, multilingualism is the norm rather than the exception’ (Wolff, 2000, p. 314).

### Multilingual settings

#### What constitutes a multilingual setting and how does it arise?

In answer to the first question, multilingual areas include both cosmopolitan villages, as they are usually called locally, and districts within cities, where the norm is the use of several languages in intra-village daily communication. Multilingual settings that have been described in the literature are shown in the map in Figure 2 and in Figure 4. Examples of such villages are Tobor, a Baïnounk village located on a national road (N4), to the north of the city of Ziguinchor, and whose multilingual situation was described by Ducos (1980). Niaguis and Agnack are cosmopolitan villages, and also originally Baïnounk villages, located on another national road (N6) to the east of Ziguinchor. Niaguis is a village located 5 to 10 km away from Agnack, one of the districts of which (Agnack Grand) was studied by Lüpke, 2010; Lüpke and Storch, 2013). In our view, districts in cities described in work on urban multilingualism in the Casamance (see Juillard, 1995; Juillard & Dreyfus, 2004) are the same types of settings as cosmopolitan villages, in that the norm is to use several languages in intra-district communication. It is important to bear in mind that village multilingualism is included in what we call societal multilingualism, but that there are other types of societal multilingualism such as district multilingualism, a concept that will not be discussed further in this study. However, a village where all or most of the speakers are multilingual is not necessarily a multilingual village. Speakers may be able to use different languages, but if they do not need to use them in the village, and they do not actually use those languages every day to facilitate communication with other villagers, we classify the village is as monolingual as long as it has one default language of communication.

To answer the subsequent portion of the question above, that being how do multilingual settings arise, we divide the multilingual settings into those which are less and more complex in their practices.

#### Simplest multilingual settings

Multilingual settings are multi-ethnic areas of migration, characterised by settlements of different linguistic communities who established themselves there at various times in the recent or remote past. These ethnic groups are peoples who identify themselves, and are identified by others, as culturally and linguistically different from the other inhabitants who live in the same setting. In the simplest cases, each community creates a settlement by occupying the same district, and speaking a variety of the language as their ‘metropole’ (Kopytoff, 1987, p. 26), i.e. the established society from where they originate. The relative homogeneity of such settlements is often reflected in the names ascribed to the districts where these migrants live. For example, district names within a village called Soon, such as Soon Jóola and Soon Mankanya, indicate that these districts are predominantly Jóola- and Mankanya-speaking. Likewise for the village of Bourofaye in which districts Bourofaye Jóola and Bourofaye Baïnounk are found, illustrating that these areas of the village were originally and predominantly inhabited by Jóola and Baïnounk speakers respectively. In terms of language use, settlers from one location may end up learning the language of another area. This is often the case with children and young people or other residents who have frequent interactions with their peers from other areas. However, it is not the case that every person in such a village becomes multilingual in all the languages spoken there.

Intra-village communication between districts in multilingual areas is generally done through local linguae francae like Mandinka, Portuguese Creole or Jóola Foñi. French may also be used in some cases, but in the last two to three decades, Wolof has been taking over as the main lingua franca in most rural settings. Sapir (1971) reports the rise of a pidgin consisting of a mix between Jóola languages Foñi and Kaasa, which was increasing as a result of speakers of related Jóola languages settling in the same area in the city of Ziguinchor. However, there is no available research on the nature of such mixed systems from different varieties of the same or related languages.

The important point to remember here is that there can be a degree of linguistic homogeneity even in multilingual rural and urban settings where some districts may be predominantly inhabited by people speaking the same language. Note however that further migration into those districts results in more complex linguistic environments, as described in the following subsection.

#### More complex multilingual settings

In more complex multilingual situations, families of different ethnolinguistic backgrounds live near each other, resulting in intense language contact between neighbours, characterised by continuous daily use of each other’s languages, and/or of a local district lingua franca. In particular, Baïnounk villages situated around the city of Ziguinchor are renowned for this type of intense multilingualism, and monolingual speakers are rare in these villages (Cobbinah, 2013; Ducos, 1980). As Ducos (1980, p. 5) argues, ‘le multilinguisme est actuellement la norme social de l’ethnie baynuk’ [Multilingualism is currently the social norm of the Baïnounk ethnic group]. Specifically, Ducos (1980) reports such a situation in Tobor; this phenomenon was also reported in Lüpke (2016b) for Agnack Grand. (Ducos, 1980, p. 6) writes:

Au niveau individuel où les situations de contacts peuvent être beaucoup plus complexes, une redondance de codes n’est pas exceptionnelle. Certains Baynuk disposent de trois, quatre, parfois cinq codes dont deux (parfois trois) peuvent avoir été acquis dans des circonstances particulières et prolongées requérant chacune leur usage; mais ces circonstances, en disparaissant, font que les codes en question n’ont plus des emplois complémentaires actuels ou à l’échelle du temps, mais concurrent.

[At an individual level where the contact situations can be more complex, [. . .] some Baïnounks have three, four and sometimes five [languages], of which two (sometimes three) may have been acquired in specific but prolonged circumstances where their use was required. But as these circumstances disappear, these languages lose their initial short- or long-term complementary functions and become languages in competition.]

The situation regarding the Baïnounk is crucially different from Mandinka, Manjack, Mankanya or Jóola groups. Jóola peoples are more numerous and dominant in the Lower Casamance. Until recently, these societies were mostly economically self-sufficient, and it was not necessary for them to be predominantly multilingual to subsist.

Ducos’s argument that certain Baïnounk people have two to three languages, which are not ‘complementary’ to Baïnounk (not simply used as lingua franca in specific situations of communication) but would actually be in competition with Baïnounk, means that it is difficult to categorise them as non-native to the individual speaker. It is important to bear in mind that this complex multilingual situation, which we label the ‘Baïnounk experience’ is by no means applicable to the rest of the Casamance (Sagna, 2016), Senegal, or the even more specific areas such as that of The Crossroads (Hantgan-Sonko, 2017). In fact, we argue in the section ‘Results’ that it only reflects the sociolinguistic reality of a minority of villages within the Casamance, which are generally located on national or other main roads, and around large cities like Ziguinchor.

It is important to bear in mind that multilingual areas may or may not be ecologies with longstanding multilingualism. The age of the settlement is not important for the typology we propose here. In other words, some villages may have been formed in precolonial times or after, and migration from various ethnic groups may have taken place separately. The key feature, however, is that among multilingual settings, immigration from people of different ethnolinguistic backgrounds has intensified in the decades that followed the independence of Senegal in 1960, resulting in increasing the rate of multilingualism (see ‘Results’ section for a detailed discussion). Thus the use of several languages at the individual and societal levels is the most important characteristic of multilingual settings. These contexts contrast with what we refer to as monolingual settings studied in the next section.

### Monolingual settings

As with our so-called multilingual settings, we seek to provide answers to the following question:

(i.a) What constitutes a monolingual setting; and (i.b) how does it differ from a multilingual setting?

To answer this question, we use concepts borrowed from canonical typology.

#### Assessing monolingualism from a canonical viewpoint

Prototype theory has been used to propose a conceptualisation of related Jóola languages as categories and to analyse multilingual data (Watson, 2019). Rather, we will use the concept of ‘canon’, as borrowed from canonical typology (Corbett, 2006, 2010), to capture the concepts of monolingualism and multilingualism as we, and our interlocutors, view the settings described here and also to help define our view on monolingualism. From a canonical perspective, as we use it here, monolingualism and multilingualism occur on a scale between the most to the least canonical. A canon is a theoretical construct that refers to the best, or the most indisputable, example of a phenomenon. In our context, this would imply finding the best example of a monolingual setting or speaker. But what would these look like? A canonical monolingual setting, if such a situation can be found, would be the extreme case of an area where one, and only one, language is spoken and no other language would be used by speakers. The speakers in such a canonical monolingual setting would be completely isolated with no contact with speakers of any other languages. This would be a case of a language with no contact with other language. However, such a situation is unrealistic. It is difficult, if not impossible, to argue for the existence of such an idealised, ‘pure’ linguistic setting exclusively composed of monolingual speakers who have no repertoire in other languages. Likewise, unless one subscribes to the opposite ideology, it would be unreasonable to argue for the existence of a canonical multilingual setting, where every single family or speaker speaks several different languages, exactly at the same level of proficiency, or where the languages that are used by individuals in that society are so mixed that speakers report that they feel it is impossible to distinguish them from one another. In other words, multilingualism, just like monolingualism, is a matter of degree. Some settings or individuals have more languages than others. The extreme cases are only points of reference from which we can assess the actual instances that are observed in real life. Thus, in this theoretical account, the canon may not actually be exemplified by entities in real life. This is where the concept of a canon differs from that of the prototype which was developed in cognitive psychology and cognitive linguistics (Croft & Cruse, 2004; Lakoff, 1987; Rosch, 1978; Taylor, 2003). Unlike a canon, one of the properties of a prototype is that it has a psychological reality in that it is represented in the minds of the speakers of a language. In this sense, if we talked about the prototypical monolingual setting or individual, we would have to show what characteristics observed in real life make it/him/her the best example to illustrate monolingualism. In the case of a canon, which is a theoretical construct, actual examples and their characteristics may not exist.

In our discussion of monolingualism in this section, we rely on the notion of a canon with the idea that neither a canonical monolingual setting (one with absolutely no contact with the outside world), nor the canonical monolingual speaker, exist. At the same time, we argue that there is no such thing as a real example of a canonical multilingual setting. However, as we show in the next subsection, the majority of rural settings in the Casamance can be referred to as monolingual settings, since, despite the presence of other languages, communication is predominantly carried out through a default language, as shown in the next section. Even when individual speakers are multilingual, one must distinguish between ability, need, and practice of multilingual communication. We will come back to these issues in the results section.

#### Monolingualism in rural Casamance

Monolingual villages of the Casamance are, like the Jóola Eegimaa-speaking area, typically mono-ethnic. Most Jóola villages, and indeed, most villages of the Casamance, are traditionally of this type (Sagna, 2016). That is, we do not find differing established linguistic communities in these settings. These settings illustrate societal monolingualism, where intra-village communication is monolingual, i.e. strongly normatively oriented towards a single default language. No linguae francae are needed within or between districts in intra-village communication. Speakers become multilingual not through daily intensive intra-village language contact, but essentially through ‘turnaround migration’ (Linares, 2003), i.e. going to the city (or other villages) and sometimes coming back. Seasonal migration among Jóola people which dates back to the 19th century (Mark, 1977) has been the main pattern of migration. According to Linares, this form of migration developed in the 1930s with the creation of cities and towns; in the decades that followed independence, rural to rural and rural to urban migration became much stronger in search of new job opportunities in sectors such as education, administration and the military, as well as jobs as housemaids (Lambert, 1994; Linares, 2003). Turnaround migration implies that migrants become more exposed to languages other than their L1. This accounts for the increase of individual multilingualism. This form of migration occurred between more ethnically and linguistically homogeneous areas to villages of migration where there are more farming opportunities, and to cities where migrants could find jobs. Migration from cosmopolitan villages or cities to more ethnolinguistically homogeneous villages such as those where Jóola people live has historically been extremely marginal, because farming opportunities are minimal. Villages of migration of the multilingual type are places where land can be bought, rented or borrowed, whereas monolingual villages have limited farming opportunities because land is normally not sold or rented to outsiders. It may be lent but that is usually for a very limited time. Land is generally owned primarily by a lineage, and secondarily by the individual who exploits the piece of land. These individuals do not have the right to rent or sell it. As Linares (2003, p. 114) argues

[A]mong the Jola of Lower Casamance, Senegal, land is abundant and inalienable; few if any persons suffer from malnutrition, let alone starvation. A person who moves elsewhere can always recover his land when and if he returns to his natal village.

The paucity of migration into monolingual areas accounts for the low level of daily language contact in these settings. Migrant families may initially use their language at home, but if they settle for a long period of time, they eventually learn the dominant local language in order to be able to communicate with other villagers, and their children end up being more fluent than their parents in the language of their environment. In the next section we present four studies that show the relevance of monolingualism in the Casamance and demonstrate how people from settings that are predominantly monolingual become multilingual.

## Materials and methods

To contextualise some of the arguments we make here, we draw on both our life and research experience in the Casamance. Sagna was born and grew up in the Casamance and has been carrying out fieldwork since 2003 in language contact, language documentation and description and child language acquisition. He also regularly moved between mono-ethnic areas such as Essil, his village of origin, and multi-ethnic areas like Baŋaaŋa and Niaguis where his family lived between 1980 – before he began primary school – and 1997 when he started university in the north of Senegal. Hantgan was a member of the Crossroads Project from 2014–2017. Her role as the project’s data manager gave her privileged insights into the Casamance context and the corpus used in the project. In addition to investigating language use in a group context, she has also begun to examine language use between members of various groups in fishing encampments along the Casamance River. She also has a good knowledge of multilingual villages like Kafountine on the north bank of the Casamance River and is also familiar with Goudomp in the east of Ziguinchor.

In terms of our study, we carried out four separate but overlapping studies, including both broad overviews of the language settings in the Casamance as well as long-term research in one specific region, namely Mof-Ávvi. Each of the studies is described in detail here, with their results provided in the following section. To ensure that our research is replicable, we provide the data in the Supplemental Materials.

### A survey of 62 villages

An initial important step to account for how people who live in a given rural area learn several languages is to discover the different kinds of ethnolinguistic groups found there, the languages that are available in their environment, and those used in their daily interactions. Such a survey must precede any generalisations about the nature of multilingualism in any area under study. This, to our knowledge, is the first attempt at such a large-scale study in the Casamance.

We undertook a survey of 62 villages from various areas of the Casamance. As part of this survey, we interviewed speakers about the sociolinguistic situation of their villages as well as villages with which they have familiarity either through living there or through regular visits. The villages used in the survey were chosen from (a) villages where our research has taken place and (b) villages from where the women in our exogamy survey came. These participants were selected from among people who were available at the time of the study and are part of an ongoing investigation concerning Casamance multilingualism.

We asked the following questions:

- How many languages are spoken in the village?
- How many ethnolinguistic groups (peoples speaking different languages or varieties, and having a different way of life) are there in the village?
- How many languages do you need to get around the village?
- Which language(s) do you speak when you go to district 1, district 2, district 3, etc.?
- When you are playing or farming with your age-mates or peers which language(s) do you use to facilitate communication?

The first question was asked bearing in mind that there may be a small settlement of people who speak a different language in a monolingual village, but use the dominant village language as a lingua franca. To these questions we add a virtual test that we call the ‘blindfold test’ which is formulated as follows:

- If you are taken blindfolded around the village, or you are walking in the dark and you hear someone coming, which language(s) will you use to greet someone and talk to them, and why?

If the speaker’s answers are ‘one language’ or ‘language X’, referring to the same language in all of these questions, the village under question is classified as a monolingual village. If more than one language is required and is used for communication to take place, than the village is categorised as a multilingual village.

### Speaker profiles

Next, we interviewed 101 speakers across four generational categories to assess their self-reported proficiency levels across languages. The data for this study were drawn from speakers’ metadata which were submitted to the Endangered Languages Archive (ELAR) at SOAS (Sagna, 2011). Although the participants’ information on their degree of multilingualism was based on their own self-reported repertoires, it is also confirmed through the first author’s personal experiences, observations and knowledge.

Note that the primary goal of the research from which the speakers’ metadata were extracted was not to study gender differences in language use, but to investigate the most endangered linguistic and cultural aspects of Eegimaa. The speakers were categorised into four age groups. Generation 4 was the most balanced group with 10 males and 10 female speakers because these speakers participated in an experimental study. Generations 1 to 3 are less balanced and contain the following male versus female ratios: 17 vs 10 for G1, 21 vs 8 for G2 and 16 vs 4 for G3. The apparent lack of balance between the groups is not due a weakness in our sampling, but to the fact that men tend to have more time to work as language consultants than women.

### Exogamy patterns and language transmission

Again in Mof-Ávvi, we studied the rate of exogamy among the 34 out of 36 women recorded as currently married in the village of Essil and living there. The other two women were not present in the village when the study took place. However, we know from personal experience that these women are from Mof-Ávvi and speak Eegimaa to their children.

### Child language acquisition: Child-directed speech and child language production

Data on child language acquisition in four villages of Mof-Ávvi come from an ongoing longitudinal study of Eegimaa-learning children starting at aged 1;10 to 4;0, in which each of the 6 children in the longitudinal group is recorded every 15 days in a naturalistic setting, interacting with multiple playmates and various caregivers. This project also includes a cross-sectional study with 10 children who are recorded once at ages 3;0 and 4;0. This is a group of children whose production is compared to the longitudinal production at ages 3;0 and 4;0. We also look at the speech of caregivers to determine what languages they use to address children.

## Results

### Village surveys: A majority of monolingual villages

The blindfold test, part of which involves speakers explaining their language choices, reveals that, while in monolingual areas speakers select one language, e.g. Bayot or Jóola Kaasa, without hesitation because, as participants report, ‘that is the language everyone uses’, in multilingual areas, speakers point out that they must know exactly where they are in the village before choosing in which language to communicate. This shows that speakers have a clear awareness of the sociolinguistic (and geographic) set-up of the villages where they live. This test also helps to establish the existence of monolingual villages in the Casamance.

**Figure 3.**
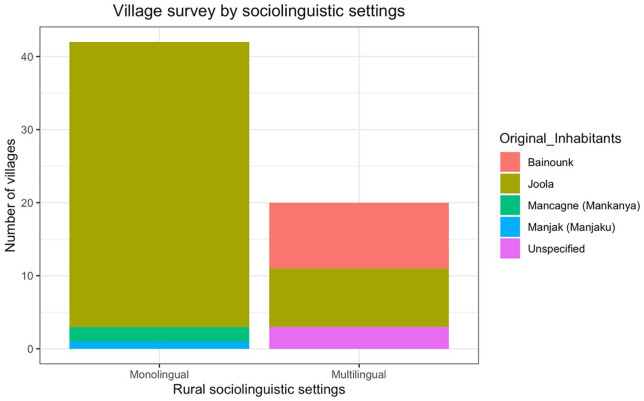
Sociolinguistic classification of villages of the Casamance according to the survey.

In peer groups from multilingual areas like Niaguis, several languages are used between peers in one age group, whereas in monolingual areas like Essil, one dominant language is used within the same age group. Additionally, the answers to the other questions in the survey allow us to assign the 62 villages surveyed into two groups: those of the monolingual and multilingual type. These results are presented in the graph and map in Figure 3.

The majority of villages in the Lower Casamance are classified by the participants in the survey as being monolingual; these villages are largely Jóola. The results in Figure 3 illustrate the responses from the survey.

**Figure 4.**
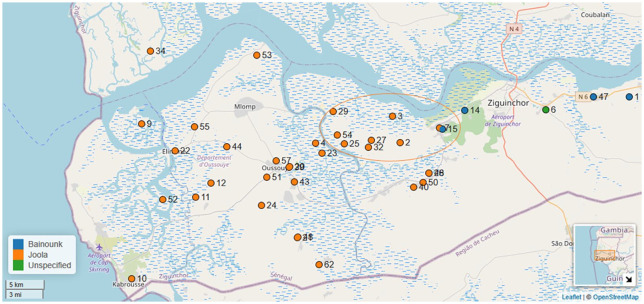
Casamance villages used in this study where the orange circle represents The Crossroads Project villages as indicated in Figure 2 (village names and associated languages are available in the Supplemental Materials). Map produced with Lingtypology package (Moroz, 2017) for R (R Core Team, 2017).

Note that the prevalence of monolingual villages in the study is not due to a sampling bias. Rather it reflects the fact that Jóola ethnolinguistic groups are dominant in the Lower Casamance. Jóola villages are, according to the results from our study, largely monolingual. These results are also displayed in the map in Figure 4.

Villages which are classified in our multilingual group are generally either Baïnounk, and/or those located on national roads, as depicted in Figures 3 and 4. These facts are important to consider for the assessment of the Casamance’s sociolinguistic situation.

Overall, we do not argue that mobility and multilingualism are recent phenomena. Rather, our overarching claim is that patterns of migration and multilingualism differ in space and time. Detailed anthropological research on the social organisation of Jóola societies in the Basse-Casamance (e.g. Girard, 1969; Schloss, 1988; Snyder, 1973; Thomas, 1959) report more homogenous settings than those described in other anthropological work (e.g. Kopytoff, 1987; Mark, 1992), which are more heterogenous. There are no written records of multilingualism in the past. Thus, any attempt to use historical generalisations to account for current multilingual situations must account for the monolingual situations, which, as our survey of 62 villages shows, turn out to be the most common ones in the Casamance. Furthermore, we stress that generalisations from one or a handful of villages (see Lüpke, 2021) cannot account for the multilingual situation in the rest of the Casamance or the broader region, e.g. Guinea. Khachaturyan and Konoshenko’s (2021) research in Guinea uses methods that are comparable to ours, e.g. a questionnaire in which they ask participants in which language would they greet a stranger. They distinguish between monolingual and multilingual settings and find that many participants in their study are ‘(quasi)-monolingual’, with limited proficiency in languages outside of their speech community.

### Speaker profiles and intergenerational decrease of monolingualism

After establishing the existence of monolingual villages in the Casamance, in the previous section the previous section on village surveys, this section, along with the following subsections on exogamy patterns and child language acquisition respectively, investigates language use in these monolingual villages at the level of the individual. We argue that the mobile younger generations of Eegimaa-speakers of Mof-Ávvi are more multilingual than older ones due to their increased exposure to other languages. The link between multilingualism and migration can be seen in Table 1, which shows the decrease in monolingualism among 101 Eegimaa speakers interviewed between 2003 and 2015. Speakers are divided into four generational groups. The span of a generational group is 20 years. Speakers from the oldest generation (G1) are those who were 70 years old and above. Those of G2 were aged from between 50 and 69, while G3 is made up of speakers between the ages of 30 and 49. The youngest generation of G4 is composed of speakers aged under 30. Note that we focus on the age variable, rather than variables like gender, since it is more relevant for showing the historical decrease of monolingualism, and leave the latter to future research.

**Table 1.** Language reporting in Mof-Ávvi.

Age range	Total	Monolingual	Percentage	Multilingual	Percentage
G1 (over 70)	27	16	59	11	41
G2 (50–69)	29	4	14	25	86
G3 (30–49)	20	0	0	20	100
G4 (under 30)	25	0	0	25	100
Total	101	20	20	101	80

Table 1 shows a decrease in monolingualism from the oldest generation 1 (G1) (59% of monolinguals) and G2 (only 14% of monolinguals), compared to G3 and G4 (no monolinguals), and a clear increase in multilingualism amongst Eegimaa speakers. This is linked to patterns of migration similar to those described by Linares (2003) for villages of three Jóola areas (Kadiamoutaye, Kalounaye, and Esudadu), where migration has increased with the younger generations, especially in the decades following independence. Such migration patterns account for the increase of individual multilingualism (or, from another viewpoint, a decrease in monolingualism) among Eegimaa speakers. This link is clearly apparent in individual speakers’ accounts of where they learned the languages they speak.

Speakers from the G1 category are mostly monolingual. Recall from the second subsection of ‘Background’ on terminological background that, in order to avoid biasing our study towards monolingualism, we included speakers who claim to have active or passive knowledge of other languages into our multilingual group. We include them in the multilingual group even when they have Eegimaa and another language for which they claim to have passive knowledge. These speakers were mostly involved in short stays away from Eegimaa-speaking areas for seasonal work (Lambert, 1994; Linares, 2003; Mark, 1977, 1978 for a discussion of these patterns of migration).

Unlike those of G1, most members of G2 have attended school in French (generally primary and secondary school), and some have lived in different places for an extended time. Speakers of G3 and G4 have spent longer periods outside Eegimaa-speaking villages, and some of them are mainly based outside. These generations of speakers include many who come back to the villages speaking Wolof as a fashionable and dating language. However, Eegimaa still remained the dominant language used with their families, villages and peer groups. Unsurprisingly, G2 to G4 participants who have been to school and spent more time living in other areas are more multilingual than the older generation G1, whose movements were more restricted than these younger generations. The languages listed by multilinguals include Jóola Kaasa, Jóola Fogny, Wolof, French and Portuguese Creole.

### Exogamy patterns: Mixed marriages do not account for multilingualism

Exogamy has been used as an argument to account for the high rate of multilingualism in the Casamance (Lüpke & Storch, 2013). We show that exogamy, by which we mean the practice of marrying outside the Eegimaa community, has a weak explanatory power on multilingualism in Mof-Ávvi. Of the 34 women we interviewed, 28 women (77%) who are married and living in Essil are from Eegimaa-speaking areas (within the Mof-Ávvi), whereas only another six women (33%) are from outside the Eegimaa-speaking area. From among the 28 married women from Eegimaa-speaking areas, eight (29%) are from Essil and 20 (71%) come from villages outside Essil. This shows that the rate of exogamy is actually much smaller in monolingual areas than has been previously stated (see Linares, 1992, p. 108 for similar findings in the Jóola Foñi area), and that endogamy (marriage within the Eegimaa community) is more the rule than the exception in Essil and in monolingual settings such as the Eegimaa-speaking area. Anthropological research carried out by Palmeri between 1975 and 1989 confirms these tendencies among the Eegimaa people. Palmeri (1995, p. 239) shows that in the village of Elubalir, 92.6% of marriages are endogamous compared to only 7.4% of exogamous marriages. This is not a peculiarity of Eegimaa-speaking villages; it is a general practice in most of rural Casamance (Linares, 1992; Palmeri, 1995). Intermarriage between members of the same communities, e.g. village or district, is possible if the partners are from different lineages, as Palmeri (1995, p. 239) shows. The argument that exogamy is a means to avoid marriage within the same gene pool does not hold since endogamy dominates.

When we examine the rate of language transmission in exogamous marriages in Essil, it appears that only two out of six mothers from outside Mof-Ávvi consistently transmit their language to their children. Two others tried but stopped, partly to avoid being mocked continuously about their language. The remaining two have not attempted to teach their language to their children. An important observation is that all the women who married in Essil have, in their own evaluation and from the knowledge the first author has of them, become fluent in Eegimaa, and the inconstant rate of transmission of mothers’ languages to their children cannot account for the high rate of individual multilingualism in Mof-Ávvi. Even if children have exposure to their mothers’ languages, they do not become multilingual as a result of exogamy, but mainly because of turnaround migration. Once again, we argue that turnaround migration, rather than exogamy, explains the current high rate of individual multilingualism in monolingual villages, whereas in multilingual villages, intra-village daily language contact accounts for the level of individual and societal multilingualism. Today, the majority of Eegimaa speakers live outside Mof-Ávvi, mainly on a permanent basis, and the rate of language transmission to new generations is in sharp decline among migrants. Within generations of speakers who have become more multilingual due to migration, there is a growing tendency to transmit only French and Wolof to children among Eegimaa migrants.

### Child language acquisition: Monolingual language transmission and production

An examination of naturalistic data taken from ongoing child language research (see Background section, subsection on child-directed speech) where recordings are made in four Eegimaa villages is presented in Table 2. The data come from three recorded files from two target children. Sanum, from the longitudinal group, was recorded at ages 1;11 and 2;0.6 (see the first column of the table) and Muna, from the cross-sectional group, was recorded at age 3;1.10. Two of Sanum’s playmates, Roga and Jandi, also participated in the recordings, and their utterances are included in Table 2. The table shows the utterances children produced during the recordings, as well as the input speech addressed to them by their mothers. The different language columns show the number of utterances produced per language. An examination of the data in the table shows very little use of languages other than Eegimaa.

**Table 2.** Child-directed speech and children’s production in Eegimaa.

File	Name	Role	Total utterances	Eegimaa	French	Wolof	Other
SAN2;0.6	Sanum	Child	49	49	0	0	0
	Jandi	Child	74	74	0	0	0
	Roga	Child	54	54	0	0	0
	Geni	Mother 1	82	82	0	0	0
	Mamia	Mother 2	176	176	0	0	0
SAN1;11.17	Geni	Mother	820	805	15	0	0
	Sanum	Child	585	585	0	0	0
	Jandy	Child	259	259	0	0	0
MUN3;1.10	Muna	Child	392	392	0	0	0

Table 2 shows the number of utterances produced by each child in the recordings, as well as child-directed speech from their mothers. Out of a total of 2491 utterances only 15 are produced in another language, French. All utterances from the children’s productions are in Eegimaa. French was used in 15 utterances by one of the mothers asking her child to repeat French greetings and songs learned at the nursery. Strictly speaking, this is not a case of child-directed speech in French as the child is not being spoken to naturally in French, but is asked to repeat words used in the context of the school.

Rare instances of the use of words other than those from Eegimaa mainly come from songs and play formulae. Until recently (less than 10 years ago), children began school at age 6 or 7. Since the introduction of nurseries among the villages of Mof-Ávvi, children are taken to the village nursery sometimes as early as age 2, for example when their mothers are working in the fields. Up to age 5, Eegimaa is the language of teaching in the nursery. In Essil, because the nursery is located within the local primary school yard, children are bound to hear some French when they start going to the school. At age 6 the children begin to be taught French as a way of preparing them for their first year in formal schooling.

Children’s early exposure to Wolof comes from trips to cities for holidays for some, and also from other children of Eegimaa descent who only speak Wolof, and who are sent to the villages to live with their grandparents or other relatives either on a permanent basis, or for a few weeks during the rainy season. Young people regularly use Wolof between themselves nowadays, as it is seen as the language of the ‘Nandités’^2^ i.e. fashionable young people; but they do not address older or younger children in Wolof mainly because the default language of communication is Eegimaa. Moreover, older people are generally reticent of speaking Wolof with other Eegimaa speakers, while children, as we point out in this section, do not speak Wolof earlier on, unless they have spent a long time in the city. While local children in our study may learn a few words of Wolof and French, the sample of data used here for illustration show that child directed speech and children production is in Eegimaa. Note that children who initially do come to the village without speaking Eegimaa end up learning Eegimaa very quickly because the language of play with their peers is Eegimaa. While it may be argued that children who have some words in Wolof or French are beginning to develop a repertoire in Wolof, the definition of multilingualism has to be significantly exaggerated for them to be categorised as multilingual.

To summarise, a broader examination of the sociolinguistic situation of rural Casamance reveals a different picture of multilingualism than the one proposed in recent work (Lüpke, 2016a, 2016b). We argue that most of rural Casamance is composed of mono-ethnic and monolingual areas. We note, however, that patterns of turnaround migration, which started before the 20th century, increasing significantly in the decades following the independence of Senegal in 1960, before turning into permanent migration in the late 1990s onwards, mean residents from monolingual areas have more exposure to other languages. This is what accounts for the sharp decrease in monolingualism across generations and the increase in individual multilingualism. Turnaround migration means that speakers from the different generations we studied here have opportunities to develop different repertoires. However, this does not mean that they do not have a default language. Results from the Crossroads Social Network Study presented by Hantgan-Sonko (2017, p. 183) revealed that only 29 out of 113 (26%) participants claim proficiency in the three languages associated with the area under study. A study of language use also clearly shows that predominance in the use of various languages differs in the individual villages Hantgan-Sonko (2017, pp. 183, 184). For example, in Essil, language use in observed communicative events is dominated by Eegimaa, while in Djibonker, Baïnounk Gubëeher is the dominant language despite a higher degree of language mixing found there. In Brin, which, as noted above, is the village at The Crossroads between Essil and Djibonker, the uses of Eegimaa and Kujireray (the Jóola language of Brin) are nearly equivalent.

## Discussion

Studies that discard the existence of monolingual settings are generally led by the perception that in the whole of the Casamance, all multilingual situations are the same. Our discussion of the different settings suggests that this is a simplistic evaluation of these sociolinguistic situations which overlooks layers of complexity created by recent movements of people from monolingual settings. In this section, we offer a brief discussion of an important methodological issue that must be addressed to provide a better understanding of the current multilingual situations in the Casamance. This is essentially an issue relating to sampling problems. Studies upon which most generalisations of the multilingual situation of rural Casamance are based were largely carried out in the same type of villages. Indeed, most of these works are concentrated among Baïnounk villages (see the first subsection of ‘Background’, on background of villages), which are known for having a much more intense form of intra-village multilingualism (Ducos, 1980).

The only village in these studies that falls into our monolingual setting is Essil (Essyl), where Goodchild carried out her fieldwork between 2015 and 2018 for her PhD research (Goodchild, 2018). Our argument that Essil and other villages of Mof-Ávvi are monolingual may be surprising, especially when one considers results presented in Goodchild (2018) where the notion of *languaging* plays an important role in the interpretation of the data. She classes Essil as part of a highly diverse multilingual area (Goodchild, 2016, p. 76) despite the absence of different ethnolinguistic groups. As shown above, societal monolingualism and individual multilingualism must be looked at separately. Our blindfold test, our examination of child language production data, as well as participant observation, have shown the default use of Eegimaa in intra-village communication in Essil, which Goodchild (2016, p. 80) also points out. Thus, monolingual language practice is still prevalent at the societal level in Essil. The canonical approach we propose here helps to make such crucial distinctions. It allows us to take into account the history of language contact of individual speakers and societies and to gain a better understanding of how multilingual repertoires develop.

The perception of Essil as a multilingual village is essentially based on a lack of use of data from Generation 1 (in our labelling). However, this should not constitute a basis for discounting monolingualism at the individual level. The fact that the last speaker of our G1 group died in 2018 indicates that individual monolingualism is not a phenomenon from a remote ancestral past. A careful examination of the participants in Goodchild’s study reveals that her oldest participants are members of our generation G2 presented in Table 1, i.e, the group with only 14% of monolinguals. G1, which is mainly composed of monolinguals, is not available to her study. We believe that in order to fully understand multilingualism in places like Essil, one must understand how it emerges across different generations. Only studying a younger population inevitably results in investigating a different population, with distinct histories of migration and contact with languages other than Eegimaa. It would be interesting, in future research, to compare data reported from The Crossroads project by Goodchild (2018) with those collected in Essil from 2003 to 2015 and beyond, especially given the growing change from monolingualism to multilingualism, and in the context of a more connected world.

In this article, we have presented completely new, rare data on immersive participant observation and a novel child language acquisition study to investigate the emergence of multilingual repertoires. We also undertook a survey of 62 villages to ascertain the existence of different kinds of monolingual/multilingual situations. These data, to our understanding, are unprecedented and the results challenge recent findings on the multilingual situations of the Casamance.

## Conclusion

The sociolinguistic context of the Casamance presents a dynamic situation, illustrating how individual multilingualism emerges in multilingual and predominantly monolingual settings. We propose an analysis of individual and societal monolingualism and multilingualism using the abstract concept of the ‘canon’ borrowed from canonical typology. This allows us to ascertain the levels of complexity in the sociolinguistic situation of the Casamance, and to account for the emergence of multilingual repertoires. The existence of monolingual settings in the Casamance has been questioned in recent research, where it is presented as mythical, based on observations made in a limited set of villages of a certain type and ethnolinguistic makeup. We surveyed 62 villages across the Casamance and argued that most villages are of the monolingual type. However, individual monolingualism has decreased across generations in those places due to turnaround migration. We studied exogamous marriage patterns in the village of Essil and its relation to intergenerational transmission and demonstrated that endogamy is the norm, and that children from these marriages become multilingual not because of multiple languages spoken at home, but because of migration. We analysed the sociolinguistic profiles of 101 Eegimaa speakers and showed that there is a strong decrease in monolingualism from the oldest generations (over 70 years old) to the youngest (under 30). A look at migratory patterns among Eegimaa people and other Jóola speakers in the last century also confirmed the strong link between migration and the decrease in monolingualism across generations. Finally, an analysis of child language data from a longitudinal study examining child-directed speech and children’s production showed that monolingual language acquisition does exist in the Casamance. Fieldworkers may often view their observations of new phenomena or places they study as ideology-free. However, unless such initial observations are supported by quantitative data analysis and a deep knowledge of their field sites, there is a danger of exhibiting an exceptionalism which does not help to elucidate the complex situations under study.

## Notes

1. Crossroads – investigating the unexplored side of multilingualism with Principal Investigator Friederike Lüpke.

2. The general meaning of ‘nandité’ is ‘someone who understands things/a clever person’. This term is used among the youth to refer to people who are thought to understand modern urban lifestyle i.e. music, fashion, etc. The language of the ‘nandité’ par excellence is Wolof.

## References

[bibr1-13670069211023146] Cobbinah A. Y. (2013). Nominal classification and verbal nouns in Baïnounk Gubëeher [PhD Dissertation]. Department of Linguistics, SOAS, University of London.

[bibr2-13670069211023146] Corbett G. G. (2006). Agreement. Cambridge University Press.

[bibr3-13670069211023146] Corbett G. G. (2010). Features: Essential notions. In Kibort A. Corbett Greville G. (Eds.), Features: Perspectives on a key notion in linguistics (pp. 141–155). Oxford University Press.

[bibr4-13670069211023146] Croft W. Cruse D. A. (2004). Cognitive linguistics. Cambridge University Press.

[bibr5-13670069211023146] Ducos G. (1980). Communautés de langue baynuk et enquêtes phonologiques. Studia Phonetica, 13, 3–8.

[bibr6-13670069211023146] Girard J. (1969). Genèse du pouvoir charismatique en basse Casamance (Senegal). IFAN.

[bibr7-13670069211023146] Goodchild S. (2016). “Which language(s) are you for?” “I am for all the languages.” Reflections on breaking through the ancestral code: Trials of sociolinguistic documentation (SOAS Working Papers in Linguistics 18), 75–91.

[bibr8-13670069211023146] Goodchild S. (2018). Sociolinguistic spaces and multilingualism: Practices and perceptions in Essyl, Senegal [PhD Dissertation]. Department of Linguistics, SOAS, University of London.

[bibr9-13670069211023146] Hantgan-Sonko A. (2017). Crossroads corpus creation: Design and case study. Yearbook of the Poznan Linguistic Meeting, 3(1), 1–32. 10.1515/yplm-2017-0009

[bibr10-13670069211023146] Juillard C. (1995). Sociolinguistique urbaine : la vie des langues à Ziguinchor (Sénégal). CNRS Editions.

[bibr11-13670069211023146] Juillard C. Dreyfus M. (2004). Le plurilinguisme au Sénégal. Karthala.

[bibr12-13670069211023146] Khachaturyan M. Konoshenko M. (2021). Assessing (a)symmetry in multilingualism: The case of Mano and Kpelle in Guinea, International Journal of Bilingualism, 25(4), 979–998.

[bibr13-13670069211023146] Kopytoff I. (1987). The internal African frontier: The making of African political culture. In Kopytoff I. (Ed.), The African frontier: The reproduction of traditional African societies (pp. 3–84). Indiana University Press.

[bibr14-13670069211023146] Lakoff G. (1987). Women, fire, and dangerous things: What categories reveal about the mind. Language (Vol. 64). The University of Chicago Press. http://www.jstor.org/stable/415440?origin=crossref

[bibr15-13670069211023146] Lambert M. C. (1994). Searching accross the divide: History, migration, and the experience of place in a multilocal Senegalese community [PhD Disseration]. Department of Anthropology, Havard University.

[bibr16-13670069211023146] Linares O. F. (1992). Power, prayer and production : The Jola of Casamance, Senegal. Cambridge studies in Social and cultural anthropology (Vol. 82). Cambridge University Press.

[bibr17-13670069211023146] Linares O. F. (2003). Going to the City . . . and coming back? Turnaround migration among the Jola of Senegal. Africa, 73, 113–132.

[bibr18-13670069211023146] Lüpke F. (2010). Language and identity in flux: In search of Baïnounk. Journal of Language Contact, 3, 155–174.

[bibr19-13670069211023146] Lüpke F. (2016a). Pure fiction – the interplay of indexical and essentialist language ideologies and heterogeneous practices: A view from Agnack. In Seyfeddinipur M. (Ed.), African language documentation: New data, methods and approaches, Special Publication No.10 of Language Documentation & Conservation (pp. 8–39). University of Hawai’i Press. http://hdl.handle.net/10125/24650.

[bibr20-13670069211023146] Lüpke F. (2016b). Uncovering small-scale multilingualism. Critical Multilingualism Studies, 4(2), 35–74.

[bibr21-13670069211023146] Lüpke F. (2021). Patterns and perspectives shape perceptions: Epistemological and methodological reflections on the study of small-scale multilingualism. Typology of Small-Scale Multilingualism, 25(4), 878–900.

[bibr22-13670069211023146] Lüpke F. Storch A. (2013). Repertoires and choices in African languages. Mouton de Gruyter.

[bibr23-13670069211023146] Mark P. (1977). The rubber and palm produce trades and the islamization of the Diola of Boulouf (Casamance), 1890–1920. Bulletin de l’Institut Fondamental d’Afrique Noire, Série B: Sciences Humaines, 39(2), 341–361.

[bibr24-13670069211023146] Mark P. (1978). Urban migration, cash cropping, and calamity: The spread of Islam among the Diola of Boulouf (Senegal), 1900–1940. African Studies Review, 21(2), 1–14.

[bibr25-13670069211023146] Mark P. (1992). The wild bull and sacred forest: Forms, meaning, and change in Senegambian initiation masks. Cambridge University Press.

[bibr26-13670069211023146] Moroz G. (2017). Lingtypology: Easy mapping for Linguistic Typology. https://cran.r-project.org/package=lingtypology

[bibr27-13670069211023146] Palmeri P. (1995). Retour dans un village diola de Casamance : chronique d’une recherche anthropologique au Sénégal. Connaissance des hommes. L’Harmattan.

[bibr28-13670069211023146] R Core Team. (2017). A language and environment for statistical computing. R Foundation for Statistical Computing.

[bibr29-13670069211023146] Rosch E. (1978). Principles of categorization. In Rosch E. Lloyd B. B. (Eds.), Cognition and categorization (pp. 27–48). Laurence Erlbaum.

[bibr30-13670069211023146] Sagna S. (2011). A digital archive of photos and over 30 hours of endangered linguistic and cultural material from the Jóola people of Mof-Ávvi (Southern Senegal). Endangered Languages Archive, SOAS.

[bibr31-13670069211023146] Sagna S. (2016). ‘Research impact’ and how it can help endangered languages. Foundation for Endangered Languages, OGMIOS, 59, 5–8.

[bibr32-13670069211023146] Sagna S. Bassène E . (2016). Why are they named after death? Name giving, name changing and death prevention names in Gújjolaay Eegimaa (Banjal). In Seyfeddinipur M. (Ed.), African language documentation: New data, methods and approaches, Special Publication No.10 of Language Documentation & Conservation (pp. 40–70). University of York. http://hdl.handle.net/10125/24652

[bibr33-13670069211023146] Sapir D. J. (1971). West Atlantic: An Inventory of the languages, their Noun Class systems and consonant Alternations. In Seboek T. A. (Ed.), Current trends in linguistics (Vol. 7, pp. 45–112). Mouton.

[bibr34-13670069211023146] Schloss M. R. (1988). The hatchet’s blood: Separation, power, and gender in Ehing social life. The University of Arisona Press.

[bibr35-13670069211023146] Snyder F. G. (1973). L’Évolution du droit foncier diola de Basse-Casamance, République du Sénégal : étude d’anthropologie juridique des rapports entre les hommes et les terres chez les Diola-Bandial [Thèse 3e cycle], Université Paris 1 Panthéon-Sorbonne.

[bibr36-13670069211023146] Taylor J. R. (2003). Linguistic categorization. Oxford textbooks in linguistics (3rd ed). Oxford University Press.

[bibr37-13670069211023146] Thomas L.-V. (1959). Les Diola : Essai d’analyse fonctionnelle sur une population de Basse-Casamance. IFAN.

[bibr38-13670069211023146] Watson R. (2015). Kujireray: Morphosyntax, noun classification and verbal nouns [PhD Dissertation], Department of Linguistics, SOAS, University of London.

[bibr39-13670069211023146] Watson R. (2019). Language as category: Using prototype theory to create reference points for the study of multilingual data. Language and Cognition, 11(1), 125–164.

[bibr40-13670069211023146] Wolff E. H. (2000). Language and society. In Heine B. Nurse D. (Eds.), African languages: An introduction (pp. 298–347). Cambridge University Press.

